# *Staphylococcus aureus* Mastitis: A Time-Course Transcriptome of Immune Activation in Small-Tailed Han Sheep

**DOI:** 10.3390/pathogens14111133

**Published:** 2025-11-07

**Authors:** Xiaoli Zhang, Li Wang, Wenzhe Chen, Xiaoyu Song, Meng Wang, Xiaojun Ma, Lijiao Yan, Chuan Wang

**Affiliations:** 1 College of Veterinary Medicine, Gansu Agricultural University, Lanzhou 730070, China; 2 Agriculture and Rural Affairs Bureau, Ruyang County, Luoyang 471400, China

**Keywords:** mastitis, *Staphylococcus aureus*, Small-Tail Han sheep, RNA-Seq, differentially expressed genes, pathological changes, pathway, inflammatory cytokines

## Abstract

Mastitis is a common mammary gland disease in mammals that severely impairs lactation function, with *Staphylococcus aureus* (*S. aureus*) being the primary pathogenic bacterium. However, the molecular mechanisms underlying *S. aureus*-induced mastitis in sheep remain incompletely elucidated. This study employed RNA sequencing (RNA-SEq) technology to systematically analyze the dynamic transcriptomic characteristics of mammary tissue in small-tailed sheep (SHT) after *S. aureus* infection, aiming to clarify the molecular regulatory mechanism of the host immune response and its relationship with the occurrence of mastitis. Twelve lactating STH were selected to establish an *S. aureus*-induced mastitis model. Blood, milk, and tissue samples were collected at 0, 24, 48, and 72 h post-infection (hpi). The infected sheep exhibited typical mastitis symptoms, including exacerbated breast swelling, reduced milk yield, elevated udder temperature, and darker, more viscous milk. Hematoxylin–eosin (HE) staining revealed significant pathological changes over time, such as stromal hyperplasia, extensive inflammatory cell infiltration, severe necrosis and sloughing of mammary epithelial cells, and compromised tissue integrity. RNA-Seq analysis identified 1299 differentially expressed genes (DEGs), among which 75 core genes maintained stable expression throughout the infection time (24 hpi, 48 hpi, and 72 hpi). Gene Ontology (GO) and Kyoto Encyclopedia of Genes and Genomes (KEGG) enrichment analyses indicated that these DEGs were associated with metabolic processes, protein binding, Toll-like receptor signaling, and the NF-κB pathway. The PPI network analysis identified core hub genes including *PTK2B*, *STAT3*, and *JAK1/3*, providing critical evidence for therapeutic target screening. Furthermore, qPCR verification indicated that the expressions of innate immune receptors *TLR2*, *TLR4*, *TLR7*, and *TLR10*, as well as pro-inflammatory factors *IL-1β*, *IL-16*, *TNF-α*, type I interferon (*IFN-α*), and nuclear transcription factor *NF-κB* were significantly upregulated in a time-dependent manner (*p* < 0.05). In conclusion, this study delineated the dynamic response of ovine mammary tissue to *S. aureus* infection, systematically elucidated temporal gene expression patterns, and revealed the molecular mechanisms underlying the tissue’s initial defense against inflammatory challenges.

## 1. Introduction

Mastitis represents a leading cause of impaired lactation in mammals. In small ruminants, both clinical and subclinical forms of this disease substantially compromise milk yield and quality. Among various mastitis-causing pathogens, *Staphylococcus aureus* (*S. aureus*) stands out as a major etiological agent responsible for severe and necrotic mastitis [1,2,3]. The pathogen expresses multiple surface proteins that mediate adhesion to and invasion of host mammary epithelial cells [4]. Upon infection, *S. aureus* is recognized by Toll-like receptors, triggering immune activation, biofilm formation, and inflammatory mediator release, ultimately leading to mammary tissue damage [5,6]. Notably, *S. aureus* can also induce subclinical mastitis in sheep, which often progresses to persistent or chronic infection, resulting in long-term production losses [7].

In addition to reducing milk output, *S. aureus* mastitis adversely affects ewes’ general health and reproductive performance, thereby threatening the sustainability of sheep farming [7]. Diminished milk production has been shown to compromise the growth of lambs [1]. Small-tailed Han sheep (STH), an indigenous Chinese breed with an average lambing rate of 280%, face a critical challenge: low lamb survival rates severely undermine farming profitability. Studies indicate that multiparous lambs frequently experience milk deficiency during early lactation, with starvation accounting for 41.7% of mortality [8]. Moreover, the rise in drug-resistant S. aureus strains not only jeopardizes lamb health and contaminates dairy products, but also poses serious risks to consumers and public health [9]. Thus, effective mastitis control is essential for enhancing livestock productivity and safeguarding human health [10]. The rising incidence of subclinical mastitis has increasingly contaminated raw milk and dairy products. In response, there is an urgent need to develop alternative strategies that reduce antibiotic use while maintaining udder health, productivity, and animal welfare standards [11,12].

Sheep represent a significant livestock species; however, the research on mastitis has predominantly concentrated on its etiology, epidemiology, diagnostic methods, and disease management, rather than molecular mechanisms [2,7,13,14]. A comprehensive understanding of the pathophysiological and molecular basis of the host response to mastitis is crucial for the development of effective control strategies. RNA sequencing (RNA-seq) has emerged as a key tool for investigating molecular mechanisms of disease pathogenesis. For example, integrative analysis of transcriptomic data has revealed extensive DNA-methylation changes that correlate with udder health and milk yield and may participate in regulating the host. And nuclear receptor subfamily 4 group A (NR4A) contributes to the ability of folic acid to attenuate high-concentration lipoteichoic acid-induced apoptosis and necrosis [15,16]. In a temporal RNA-Seq study of *E. coli*-infected dairy sheep mammary glands, LPS-induced inflammation and milk synthesis pathways were suppressed, while neutrophil recruitment and T cell/interferon responses rose then declined between 6 and 24 h, elucidating the molecular trajectory of mammary innate immunity [17]. In addition, the protein and RNA expression profiles of bovine mammary epithelial cell line MAC-T infected with *S. aureus* have been elucidated [18,19]. However, the above-mentioned studies cannot adequately reflect the highly complex pathological changes occurring during actual mammary infection, nor can they simulate the biologically significant cell–cell interactions that occur in vivo. The transcriptional expression profiles to *S. aureus* infection in ovine mammary glands have not been fully elucidated. Therefore, to clarify the mechanisms of the inflammatory response in ovine mammary tissue following *S. aureus* infection and the molecular interaction network between epithelial and immune cells, this study employed RNA sequencing to systematically analyze the dynamic transcriptomic landscape of the mammary gland in STH post-infection. Our results reveal intrinsic connections among inflammatory progression, immune response dynamics, and gene expression reprogramming. This research establishes a solid theoretical foundation for deciphering host immune regulation mechanisms, uncovering the pathogenesis of mastitis, and identifying key molecular targets for *S. aureus* mastitis intervention.

## 2. Materials and Methods

### 2.1. Bacterial Growth Conditions

*S. aureus* was isolated and identified from milk samples collected from sheep with clinical mastitis at a commercial sheep farm in Tianzhu County, Gansu Province. *S. aureus* was cultured on Tryptic Soy Broth (TSB) (Beijing Solarbio Science & Technology, Beijing China) agar plates and incubated overnight at 37 °C. Individual colonies were then transferred to 2 mL of liquid TSB medium and cultured for 12 h at 37 °C in a rotary shaker. Subsequently, 100 μL of the bacterial culture was added to sterile TSB at a 1:100 dilution and incubated at 37 °C with shaking until the optical density at 600 nm (OD_600_) reached 1.0. Finally, the bacterial suspension was serially diluted in DMEM to achieve a concentration of approximately 3 × 10^3^ CFU/mL.

### 2.2. Establishment of a Model of Mastitis Induced by S. aureus in STHS

Twelve primiparous STH in mid-lactation were obtained from a large-scale farm in Dingxi, Gansu Province. All animals were approximately 2 years old, clinically healthy, and had no history of mastitis. These sheep were housed in freestall pens at the Experimental Animal Center of Gansu Agricultural University, fed the same diet, and given free access to water. They were milked twice daily (once in the morning and once in the afternoon), and the milk production volume of each mammary chamber was recorded every day. Before the experiment, the STH underwent a comprehensive examination. Sterilized test tubes (Becton, Dickinson and Company, USA) were used to collect milk samples for bacterial testing and physicochemical analysis. Only sheep with normal body temperature, normal udder appearance (no swelling, heat, or hard lumps), negative LMT results, and negative bacterial culture in milk were eligible for the challenge test.

The STH were randomly divided into two groups: 9 in the experimental group and 3 in the control group. Before bacterial challenge, the teat orifice was disinfected with alcohol swabs. The udders were then washed with warm water, and the milk was fully expressed. A sterilized small-diameter infusion cannula (or lactation needle) (Becton, Dickinson and Company, Franklin Lakes, NJ, USA) was inserted into the teat canal to drain any residual milk. Next, 0.5 mL of bacterial suspension was drawn into the cannula and infused into the udder, followed by gentle massage for several seconds. The contralateral udder half was disinfected and injected with the same volume of sterile saline (Beijing Solarbio Science & Technology, Beijing China) as in the control group. Each animal received one single intramammary challenge. No repeated challenge or re-infection was performed.

### 2.3. Clinical Symptom Monitoring and Sample Collection

The activity status of the STHs was evaluated through behavioral observations and response tests. Under natural light conditions, udder color changes were carefully monitored. The degree of udder swelling was quantified by measuring teat-thickness differences using vernier calipers (Guanglu Measuring Instrument Co., Ltd., Guilin, China) Milk yield was precisely determined using a standard milking cup coupled with an electronic scale (KaiFeng Scales, Jinhua, China). Milk consistency was assessed through the sloshing test method. Results were recorded and categorized into three severity grades: mild (+), moderate (++), and severe (+++).

Mammary tissue samples were collected from the inoculated udder quarters of the STH at 24 h, 48 h, and 72 h post-inoculation, as well as prior to inoculation (0 h) [20]. The sheep were anesthetized using chloral hydrate. Following anesthesia, the mammary glands were aseptically prepared using alcohol and iodine solution. Under sterile conditions, a sagittal incision was made, and tissue blocks (0.5 cm × 0.5 cm × 0.5 cm) were excised from the mammary gland. After sample collection, the sheep were humanely euthanized.

### 2.4. Histopathological Examination

The mammary tissues were fixed in 4% formaldehyde solution and embedded in paraffin to prepare 4 μm thick sections. Tissue sections were stained with hematoxylin and eosin (H&E) following standard protocols. Lesion areas of interest were then photographed and analyzed using a Leica DM750 microscope (Leica Microsystems GmbH. Wetzlar, Germany) imaging system.

### 2.5. RNA Extraction and RNA Sequencing

Total RNA was extracted from the collected mammary tissue samples using the RNeasy kit (Vazyme R401-01) according to the manufacturer’s protocol. Briefly, tissues were pulverized in liquid nitrogen and homogenized in lysis buffer containing high-concentration salt ions, followed by phenol–chloroform extraction. RNA integrity was verified by 1% agarose gel electrophoresis and Agilent 2100 Bioanalyzer analysis, with all samples showing RNA integrity numbers (RINs) between 9.5 and 10. Poly (A)+ RNA was enriched from total RNA using oligo(dT)-attached magnetic beads. First-strand cDNA was synthesized by reverse transcription, followed by second-strand synthesis, end repair, and adapter ligation. The cDNA library was amplified by PCR and quantified using a Qubit 2.0 Fluorometer. Finally, paired-end sequencing (150 bp) was performed on the Illumina NovaSeq 6000 platform [21,22].

### 2.6. Summary of the RNA-Seq Data and Bioinformatics Analysis

High-throughput sequencers generate image data of sequencing fragments, which are converted into sequence reads through CASAVA base calling. These reads contain both nucleotide sequence information and corresponding quality scores. We analyzed sequencing error rate distribution and measured GC content across all samples. Raw sequencing data were quality-filtered using fastp (v0.19.7), followed by evaluation of filtered data quality. The reference genome (Bos_taurus_Ensembl_104) was indexed using HISAT2 (v2.0.5), and paired-end clean reads were subsequently aligned to the reference genome using HISAT2 (v2.0.5) [21,22]. An average of 56,696,470, 52,631,676, 47,984,998, and 45,485,796 high-quality clean reads were obtained from STH samples at 0 h, 24 h, 48 h, and 72 h post-infection, respectively (Supplementary Table S1). Using an FPKM cutoff value > 0.01 to identify potentially expressed genes, we detected averages of 2280 (0 h), 7862 (24 h), 12,446 (48 h), and 3248 (72 h) expressed genes in *S. aureus*-infected STH mammary tissues. Through differential expression analysis (criteria: |log2(fold change)| ≥ 1 and FDR-adjusted q-value ≤ 0.05), we identified 1299 significantly differentially expressed genes (DEGs) across the infection time course. These findings provide valuable data for subsequent mechanistic studies of *S. aureus* mastitis pathogenesis.

### 2.7. Differential Expression Analysis

Transcript and gene expression levels were quantified and comparatively analyzed between uninfected and *S. aureus*-infected mammary tissues. Differential expression analysis was performed using the DESeq2 package (v1.20.0) in R, with statistical significance thresholds set at an adjusted *p*-value < 0.05 and an absolute log2 fold change > 1. These stringent criteria identified biologically relevant differences in gene expression patterns associated with mastitis infection.

### 2.8. GO and KEGG Enrichment Analysis

Gene Ontology (GO) enrichment analysis of differentially expressed genes (DEGs) was performed using the clusterProfiler R package (v3.8.1) with gene length bias correction. The same package was employed to analyze statistical enrichment of DEGs in KEGG pathways.

### 2.9. PPI Analysis of DEGs and Clustering Analysis of DEGs

Protein sequence alignment was performed using DIAMOND software (v0.9.13) to compare the target gene sequence (NCBI accession: NC_056054.1) against reference protein sequences. Subsequently, an interaction network was constructed based on known protein–protein interactions (PPIs) from the reference species.

### 2.10. RT-qPCR

To validate the RNA-seq results, four candidate genes were randomly selected for relative expression analysis using RT-qPCR. Gene-specific primers were designed for each target (Supplementary Table S2). To investigate the inflammatory response induced by *S. aureus* infection in STH mammary tissues, cytokine expression patterns were analyzed at multiple time points (12, 24, 36, 48, and 72 hpi). Total RNA was extracted from cultured cells using TRIzol Reagent (Life Technologies Corporation, Carlsbad, CA, USA)) and reverse transcribed into cDNA following the manufacturer’s protocol. Quantitative PCR was performed using SYBR Premix Ex Taq II (TaKaRa Bio., Shiga, Japan) on a QuantStudio 5 Real-Time PCR System (Applied Biosystems, Waltham, MA, USA) with the following thermal cycling conditions: initial denaturation at 95 °C for 5 min, followed by 40 cycles of denaturation at 95 °C for 30 s, annealing at 58 °C for 30 s, and extension at 72 °C for 30 s. *β-actin* was used as the internal reference gene for normalization. Relative mRNA expression levels were calculated using the 2^−ΔΔCt^ method. All reactions were performed in technical triplicates, and melting curve analysis was conducted to verify amplification specificity.

### 2.11. Statistical Analysis

RT-qPCR experimental data were presented as (means ± SD). Analyses were performed by using GraphPad Prism Software 8.0 (San Diego, CA, USA) and IBM SPSS Statistics version 28.0.1 (IBM Corp., Armonk, NY, USA). Student’s *t*-test was used for comparing two groups. And the log-rank test was used to calculate *p*-values. * *p* < 0.05, ** *p* < 0.01, and *** *p* < 0.001 were considered statistically significant.

### 2.12. Ethics Statement

This study strictly complied with national and institutional ethical regulations and guidelines governing animal experimentation. Throughout the experimental procedures, all possible measures were implemented to minimize pain and discomfort in the STH. Surgical interventions were performed under chloral hydrate anesthesia to ensure the absence of intraoperative pain. In accordance with experimental protocols and ethical standards, subjects were humanely euthanized at the study’s conclusion using approved euthanasia methods to prevent unnecessary suffering.

These measures guaranteed that all animal experiments adhered to principles of scientific rigor, methodological validity, and ethical integrity. All procedures involving dairy sheep were approved under protocol GAU-LC-2020-32.

## 3. Results

### 3.1. Clinical Signs and Symptoms

In this study, an in vivo model of *S. aureus*-induced mastitis was successfully established in STH through intramammary challenge. Compared to the control group, the infected STH exhibited progressively reduced activity, decreased feed and water intake, elevated body temperature, intensified swelling in the mammary gland region, and diminished milk production as infection duration increased. The milk also showed a darkening in color and increased viscosity (Table 1). These clinical manifestations confirmed the successful establishment of the mastitis model, providing a foundation for subsequent analyses.

### 3.2. Morphologic Changes in Breast Tissue After S. aureus Infection

**Figure 1:**
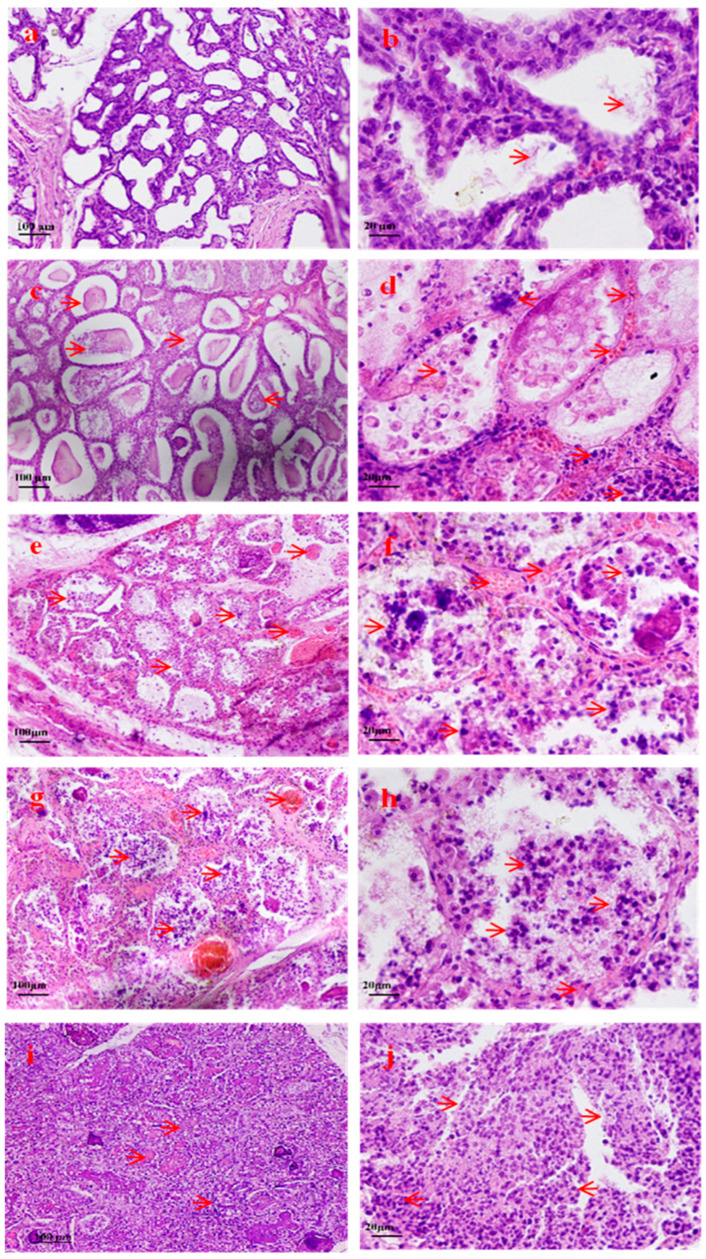
Morphological changes in breast tissue after *S. aureus* infection. (**a**) The mammary tissue showed maintained normal structure and intact acini (H.E × 100). (**b**) The epithelial cells of the mammary acini were neatly arranged, and milk (red arrow) was observed in the lumina (H.E × 400). (**c**) Mild interstitial edema was present in the mammary gland, with increased plasma effusion observed in the acinar lumen(red arrow) (H.E × 100). (**d**) The mammary acinar epithelium demonstrated focal detachment(red arrow) (H.E × 400). (**e**) Extensive inflammatory cell infiltration was observed in the interlobular stroma, perivascular areas, and interacinar spaces (red arrow) (H.E × 100). (**f**,**g**) The mammary stroma exhibited widening with hemorrhagic edema (red arrow) (H.E × 400 and H.E × 100). (**h**) The mammary acinar epithelium disintegrated, leading to a predominance of neutrophils (red arrow) (H.E × 400). (**i**) Mammary acini showed epithelial necrosis and detachment, characterized by luminal fusion and architectural collapse (red arrow) (H.E × 100). (**j**) Dense inflammatory infiltration led to the near-total loss of the mammary tissue architecture (red arrow) (H.E × 400).

The pathological changes in mammary tissue in small-tailed Han sheep after *S. aureus* infection were studied by the HE-staining method. In the control group (Figure 1a,b), the mammary tissue maintained normal histoarchitecture with intact glandular follicles, neatly arranged epithelial cells, and visible lactating vesicles. At 24 hpi, the infected group exhibited mild interstitial edema, increased plasma exudation into the glandular lumina, and focal detachment of the follicular epithelium (Figure 1c,d). At 48 and 72 hpi, pathological features progressed to extensive inflammatory cell infiltration (interlobular stroma, perivascular areas, and alveolar spaces), epithelial disintegration with neutrophil/granulocyte predominance, stromal widening with hemorrhagic edema, persistence of lactating vesicles amidst inflammatory changes (Figure 1e–h). At 96 hpi, the samples demonstrated severe pathological manifestations: necrotic epithelial detachment, alveolar fusion and structural collapse, dense inflammatory infiltrates, and near-total loss of tissue organization (Figure 1i,j). Following inoculation, *S. aureus* proliferated within the mammary glands of STH, inducing distinct pathological changes.

### 3.3. Identification of DEGs

**Figure 2:**
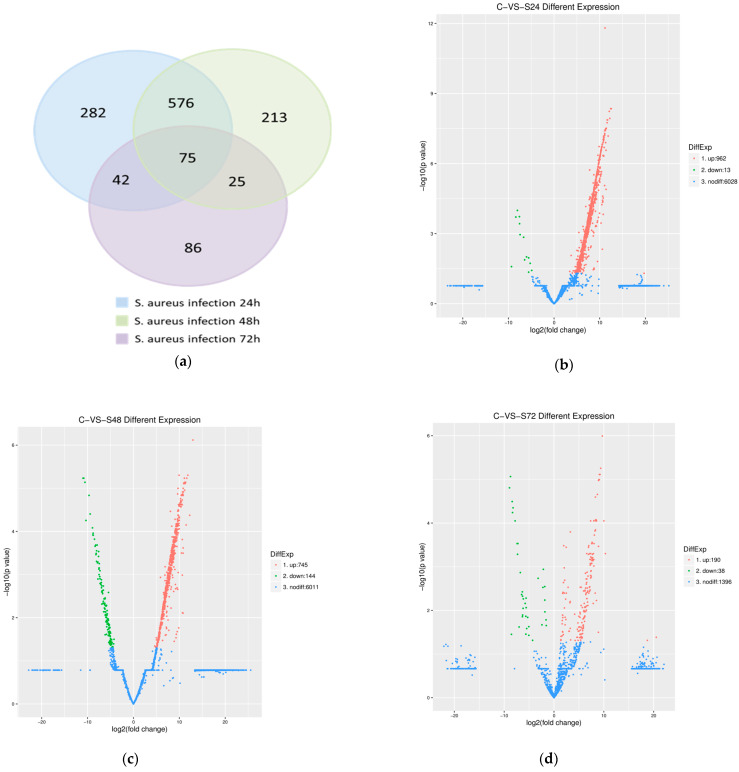
Distribution of DEGs in STHS breast tissues at different time periods of *S. aureus* infection. (**a**) Venn diagram summarizing the number of genes expressed only at 24 h, 48 h, and 72 h, and shared with each other. (**b**–**d**) Volcano plots of changes in DEG expression at 24 h, 48 h, and 72 h. Red and green dots indicate genes that were upregulated and downregulated in STHS mammary glands compared to infected 0 h mammary tissue, respectively (*p* < 0.05). Blue dots represent genes with no significant difference (*p* > 0.05).

Transcriptomic analysis identified 1299 significant differentially expressed genes (DEGs) during *S. aureus* infection across three time points (Figure 2a), revealing a temporal pattern of gene regulation: at 24 hpi, 975 DEGs were detected (962 upregulated, 13 downregulated; Figure 2b), and this decreased to 889 DEGs (745 upregulated, 144 downregulated) at 48 hpi (Figure 2c), and further declined to 228 DEGs (190 upregulated, 38 downregulated) by 72 hpi (Figure 2d). The results indicated that as the infection process progressed, the overall transcriptional activity of the host gradually weakened, and the direction of gene expression regulation changed from being dominated by downregulation to a significant increase in the proportion of downregulated genes. Notably, time-specific DEGs were observed, with 281, 213, and 86 genes uniquely expressed at 24, 48, and 72 h, respectively. Comparative analysis using the DESeq method identified that 75 core DEGs were expressed at all time points (Figure 2a). Additionally, KEGG pathway enrichment analysis revealed temporally dynamic pathway activation patterns, with the highest number of enriched pathways (224) observed at 24 hpi, followed by 221 pathways at 48 hpi, and a marked reduction to 150 pathways by 72 hpi. This dynamic pattern demonstrates that as the infection progresses, the host immune response transitions from broad-spectrum nonspecific defense mechanisms to more precisely targeted specific immune defenses.

### 3.4. Gene Ontology (GO) Enrichment Analysis of the DEGs

**Figure 3:**
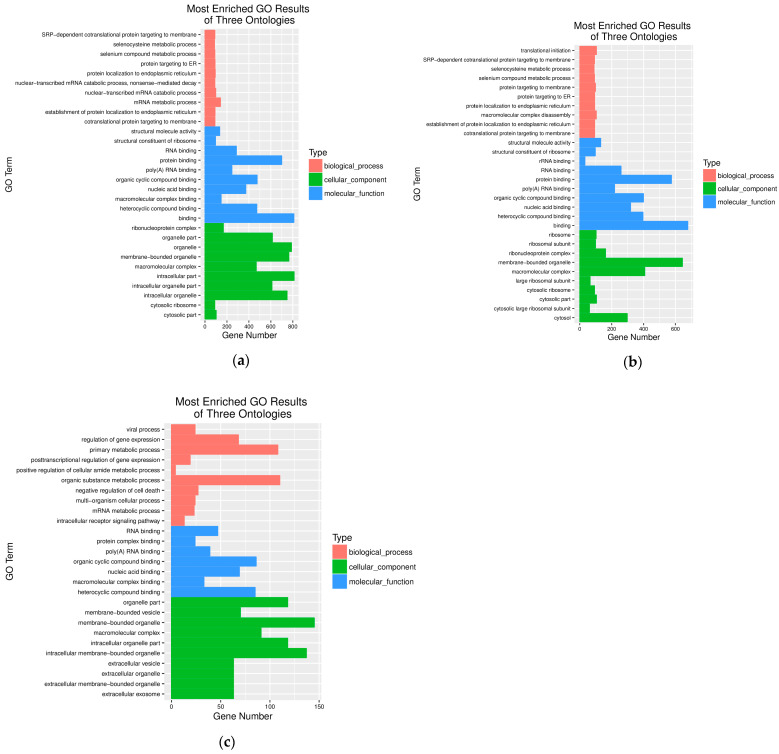
GO classification of differentially expressed genes at different infection time periods: (**a**) At 24 h after infection, (**b**) at 48 h after infection, and (**c**) at 72 h after infection. The top 10 GO terms are enriched in biological processes, cellular components, and molecular functions.

At 24 h post *S. aureus* infection of STH mammary tissue and after GO enrichment analysis, the most significantly enriched terms were intracellular organelle, membrane-bounded organelle, organelle, and intracellular part in the cellular component (GO-CC) category. For molecular function (GO-MF), the predominant terms were organic cyclic compound binding, protein binding, and general binding activity. Within biological processes (GO-BP), mRNA metabolic process emerged as the most enriched term (Figure 3a). At 48 hpi, GO enrichment analysis revealed significantly enriched terms included macromolecular complex, membrane-bounded organelle, and cytosol in the cellular component (GO-CC) category. For molecular function (GO-MF), the predominant terms were heterocyclic compound binding, protein binding, and general binding activity. Within biological processes (GO-BP), key enriched terms included translational initiation, protein targeting to membrane, and macromolecular complex disassembly. By 72 hpi, enriched processes were primarily associated with regulation of gene expression, primary metabolic process, organic substance metabolic process, and negative regulation of cell death in GO-BP. The GO-CC category showed enrichment in organelle-related components, while GO-MF was predominantly enriched in the RNA binding pathway (Figure 3c). The temporal analysis demonstrated a progressive increase in both the number and complexity of DEGs in GO-BP categories as infection duration extended (Figure 3c). This pattern suggests a dynamic host response that evolves from basic cellular processes to more complex regulatory mechanisms during prolonged *S. aureus* infection.

### 3.5. KEGG Pathway Analysis of the DEGs

**Figure 4:**
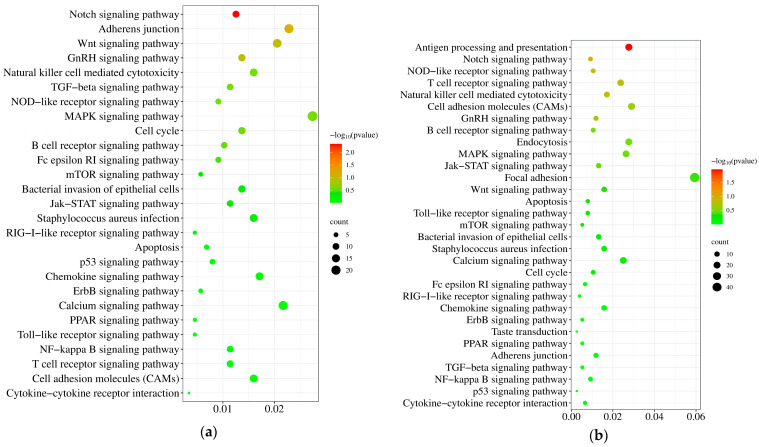

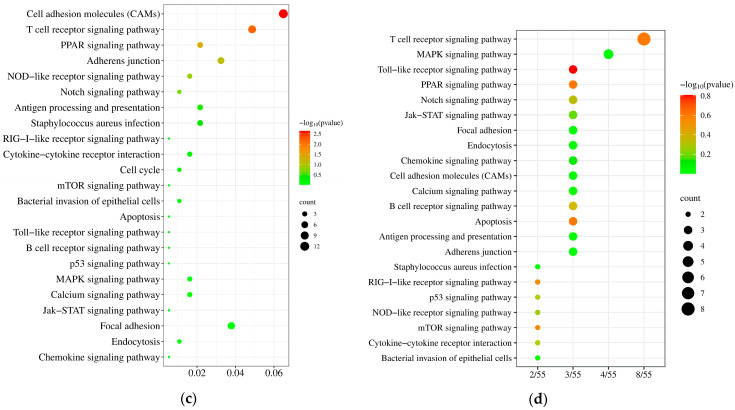

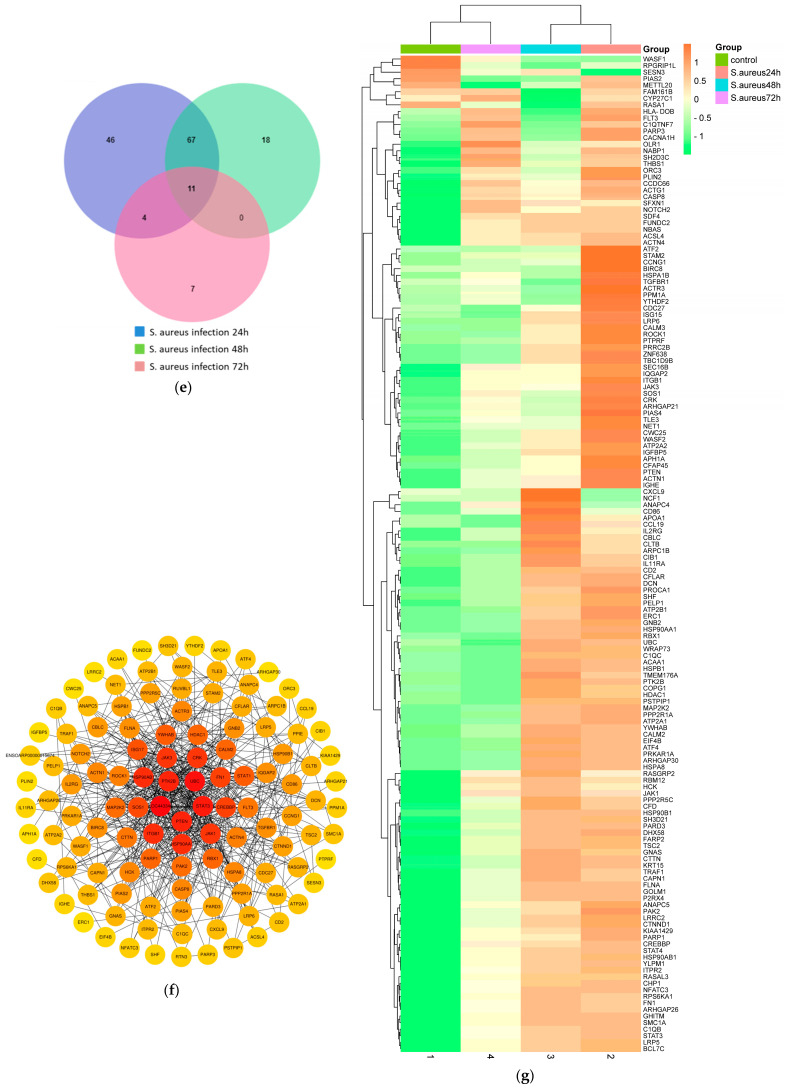
Analysis of screening KEGG enrichment pathways related to inflammation and apoptosis at different infection time periods: (**a**) At 24 h after infection, (**b**) at 48 h after infection, and (**c**) at 72 h after infection. (**d**) Screen out of the same immune- and apoptosis-related signaling pathways in different time periods. (**e**) DEGs of immune- and apoptosis-related pathways in different time periods. (**f**) PPI of selected DEGs. (**g**) Heatmap of the relative expression levels of selected DEGs in the 24 h, 48 h, and 72 h infection groups. Fold changes in expression collapsed on a color scale, with orange blocks represent increased DEGs, while green blocks indicate suppressed DEGs.

KEGG pathway analysis revealed temporally dynamic changes in inflammation- and apoptosis-related signaling pathways during *S. aureus* infection of STH mammary tissue. At 24 hpi, 30 pathways involving 128 DEGs were significantly enriched, with the MAPK signaling pathway, cell adhesion molecules (CAMs), and calcium signaling pathway being most prominent (Figure 4a). At 48 hpi, pathway enrichment persisted but decreased to 31 pathways (96 DEGs), showing sustained activation of focal adhesion, MAPK signaling, and CAMs (Figure 4b). At 72 hpi, only 23 pathways (22 DEGs) remained enriched, predominantly featuring T cell receptor signaling along with continued CAMs and focal adhesion activity (Figure 4c), demonstrating a progressive refinement of the immune response over time.

We analyzed 155 differentially expressed genes (DEGs) across 22 inflammation- and apoptosis-related signaling pathways (Figure 4d,e). Gene expression levels were represented by FPKM values, with distinct color regions indicating different clustering groups. Clustering analysis revealed temporal expression variations, demonstrating that identical genes exhibited differential expression patterns among the 24, 48, and 72 hpi groups (Figure 4g). The hierarchical clustering analysis of the DEGs is presented in Figure 4g. Subsequently, protein–protein interaction (PPI) analysis of these 155 DEGs identified a network comprising 120 interconnected nodes. The identified hub genes including *PTK2B*, *UBC*, *STAT3*, *JAK1*, and *JAK3* exhibited the highest network connectivity, strongly suggesting their central regulatory roles in mediating the host inflammatory response to *S. aureus* infection in mammary tissue (Figure 4f).

### 3.6. Validation of RNA-Seq Results Using an RT-qPCR Approach

**Figure 5:**
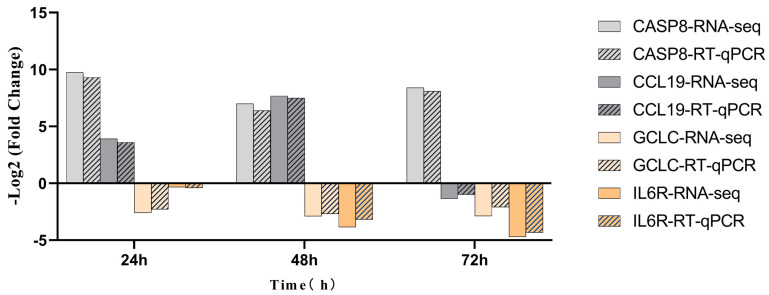
Validation of the differentially expressed genes by RT-qPCR.

For validation, we randomly selected four DEGs (*CASP8*, *CCL9*, *GCLC*, and *IL6R*), including two upregulated genes (*CASP8* and *CCL9*) and two downregulated genes (*GCLC* and *IL6R*). Notably, *CCL19* showed downregulation at 72 h post-infection. We normalized all expression levels to *β-actin*, which demonstrated stable expression under our experimental conditions. The RT-qPCR results (Figure 5) confirmed the accuracy of the RNA-Seq data for quantifying gene expression in STHS mammary gland parenchyma.

### 3.7. Detection of Expression of Inflammatory Factors at Different Stages of Infection

**Figure 6:**
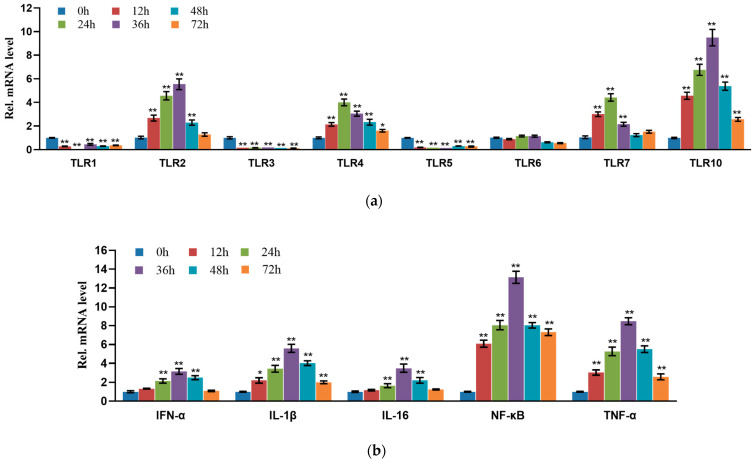
Validation of inflammation and immune-related gene expression levels: (**a**) Toll receptor-related gene expression levels and (**b**) expression levels of *IL-1β*, *IL-6*, *TNF-α*, *IFN-α*, and *NF-κB*. The independent experimental group (n = 5) underwent sampling at 0, 12, 24, 36, 48, and 72 h post-infection. * *p* < 0.05, ** *p* < 0.01.

To investigate immune response activation in STH mammary glands following *S. aureus* infection, we performed systematic analysis of key immune-related molecule expression patterns using RT-qPCR. The results revealed distinct temporal expression profiles of Toll-like receptors (TLRs), showing that *TLR2*, *TLR4*, *TLR7* and *TLR10* were significantly upregulated in a parabolic pattern, with *TLR2* and *TLR10* peaking at 36 h while *TLR4* and *TLR7* reached maximum expression at 24 h post-infection (Figure 6a). In contrast, *TLR1*, *TLR3* and *TLR5,* expression was significantly downregulated, whereas *TLR6* remained unchanged throughout the infection period. Furthermore, we observed that pro-inflammatory mediators including *IFN-α*, *IL-1β*, *IL-16*, *NF-κB,* and *TNF-α* exhibited similar dynamic patterns, with their expression levels progressively increasing during early infection, peaking at 36 h, and subsequently declining with prolonged infection time (Figure 6b). These findings demonstrate that *S. aureus* infection triggers a time-dependent immune response in STHS mammary glands, characterized by coordinated activation of both pattern recognition receptors and downstream inflammatory mediators.

## 4. Discussion

Mastitis is an inflammatory disease of the mammary gland caused by bacterial and other microbial infections. As a multifactorial disease, mastitis poses substantial threats to both animal welfare and dairy production systems, with severe cases incurring significant economic burdens on global livestock industries [1,2,3]. Among the diverse etiological agents, *S. aureus* emerges as a particularly problematic pathogen in ruminant mastitis, owing to its remarkable capacity for recurrent infection and frequent transition to chronic states that resist conventional therapies [3,5]. The clinical progression and pathological severity of *S. aureus*-induced mastitis appears intrinsically linked to strain-dependent variations in virulence determinants, facilitating intracellular persistence within mammary epithelial cells, which is a key mechanism enabling immune evasion and therapeutic resistance [23,24]. The escalating prevalence of antimicrobial resistance, exacerbated by widespread antibiotic misuse, has significantly complicated the clinical management of *S. aureus* mastitis. While bovine mastitis has been extensively studied, transcriptomic investigations of *S. aureus* infection in ovine models remain limited. Our study provides the first comprehensive transcriptomic analysis of *S. aureus* infection dynamics in ovine mammary tissue, addressing this critical knowledge gap and complementing existing research on mastitis prevention and treatment.

Our study successfully established an in vivo *S. aureus* mastitis model in ovine mammary tissue, enabling comprehensive temporal transcriptomic analysis. Across four infection time points (0, 24, 48, and 72 h), we identified 1299 significant differentially expressed genes (DEGs) (Figure 2a). Differential expression analysis using DESeq2 identified 75 genes with stable expression patterns throughout the entire infection course (FDR < 0.05), suggesting their potential core regulatory functions in host–pathogen interactions. Among the persistently expressed DEGs, two well-characterized apoptosis regulators were identified: *CASP8* (caspase 8), the principal initiator of extrinsic apoptosis pathways that activates downstream caspase cascades, and *BAG6* (BCL2-associated athanogene 6), which critically modulates apoptotic signaling through interactions with *BCL2* family proteins. The remaining differentially expressed genes (Supplementary Table S3), though currently uncharacterized or poorly studied, may represent novel components of mammary epithelial cell defense mechanisms, previously unrecognized bacterial virulence targets, or potential regulators of immune cell recruitment and activation during *S. aureus* infection. Our findings reveal significant disruptions to normal mammary gene expression patterns during *S. aureus* infection. While previous lactation studies in sheep have identified *EEF1A1* and *RPS29* as highly expressed, stable reference genes across lactation stages [25,26], and casein genes (*CSN1S1*, *CSN1S2*, *CSN2*, *CSN3*) along with lactalbumin genes (*LALBA*, *LGB*) as markers of mammary function [27], our transcriptomic analysis yielded several notable observations: *CSN3* showed initial upregulation at 24–48 hpi followed by complete absence at 72 hpi, while *EEF1A1*, *RPS29*, and other casein and lactalbumin genes (expected lactation markers) were undetectable among the differentially expressed genes. The widespread suppression of these critical genes is consistent with the significant decline in milk yield observed in STH at 48–72 hpi. These findings demonstrate *S. aureus* infection induces rapid downregulation of lactation-related genes, progressive failure of milk synthesis machinery, and fundamental physiological changes in mammary epithelium. The disappearance of *CSN3* and absence of other milk protein genes provide molecular evidence for clinical lactation failure. This widespread transcriptional downregulation likely underlies the lactation failure characteristic of *S. aureus* mastitis, reflecting both direct pathogen-mediated disruption and host resource reallocation toward immune defenses.

Our transcriptomic analysis identified two key genes *CASP8* (caspase 8) and *APOE* (*apolipoprotein E*) that were significantly upregulated during *S. aureus* infection in STH mammary tissue, revealing complex host–pathogen interactions. As the initiator of extrinsic apoptosis, *CASP8* activation initiates a proteolytic cascade [28]: the zymogen *caspase-8* undergoes proteolytic processing to form activated *caspase-8* (cleaved caspase-8, p18/p10), which in turn cleaves and activates *caspase-3* (cleaved caspase-3, p17/p12). This sequential activation cascade subsequently modulates *Bcl-2* family proteins, inducing pro-inflammatory apoptosis that ultimately contributes to inflammation resolution [29]. Notably, we observed concurrent activation of *BAG6*, a *Bcl-2* family member, suggesting a sophisticated immunoregulatory mechanism whereby *S. aureus* infection simultaneously induces inflammatory responses through pathogen recognition while activating *BAG6*-mediated counter-regulatory pathways to prevent excessive inflammation. Furthermore, *caspase-8* mediates suppression of pro-inflammatory cytokines (*IL-1β*, *IL-6*, and *TNF-α*) through its inhibitory effects on both the *NLRP3* inflammasome and *IFN-I* signaling pathways [30,31], collectively demonstrating that these pathways maintain a critical balance between pro- and anti-inflammatory responses, which dictates the severity of the acute infection. The *APOE* gene encodes apolipoprotein E, a key lipid-binding protein that primarily supplies cholesterol precursors for steroid hormone biosynthesis, including ovarian estrogen and progesterone production [32]. In STH mammary tissue, we observed distinct *APOE* expression patterns under different physiological states: while showing high expression during non-lactating periods (potentially contributing to the breed’s characteristic high fertility through enhanced steroidogenesis), its expression was markedly suppressed during lactation. Intriguingly, *S. aureus* infection induced significant *APOE* upregulation in mammary tissue, followed by a progressive decline with prolonged infection duration, suggesting a potential biphasic response involving initial metabolic adaptation and subsequent pathological dysregulation during mastitis progression.

RNA-seq results revealed that the number of DEGs was significantly lower at 72 h than at 24 h and 48 h as the infection progressed. The underlying mechanisms of this phenomenon require further investigation. Additionally, we observed significant upregulation of the pro-inflammatory cytokine/chemokine Gasdermin D (*GSDMD*)—a cysteine aspartase substrate and member of the Gasdermin family implicated in immune responses—at 24 h and 48 h, but it was undetectable at 72 h. Previous studies have demonstrated that *GSDMD* mitigates *S. aureus* skin infections by suppressing Cxcl1-Cxcr2 signaling and plays a critical role in protecting skin tissues during infection [29]. In summary, persistent *S. aureus* infection might be able to disrupt the STH’s own immune protection mechanism, leading to the development of mastitis.

Previous studies have established the central role of cellular oxidative detoxification and redox processes in *E. coli*-induced mastitis, identifying 10 core genes as potential candidates for disease prevention and early diagnosis [33,34]. Notably, similar oxidative stress pathways were observed in *S. aureus*-induced subclinical mastitis [35], highlighting their fundamental importance in mammary gland inflammation. Our temporal GO analysis revealed dynamic progression of biological responses: during early infection (24–48 h), processes were predominantly enriched in protein targeting to membrane- and SRP-dependent cotranslational protein targeting (GO-BP), membrane-bound organelles and cytosol (GO-CC), along with structural molecule activity and RNA binding (GO-MF). This pattern reflects a rapid emergency response by the host to pathogen invasion, aimed at maintaining cellular integrity and secreting defense factors. Clinically, this phase corresponds to mild functional disturbance and initial inflammatory cell infiltration in the mammary gland. At 72 hpi, the response shifted substantially toward regulation of gene expression, primary metabolic processes, and negative regulation of cell death (GO-BP), suggesting a transition from initial stress responses to more complex regulatory mechanisms as the infection progressed. Our findings demonstrate that prolonged *S. aureus* infection exacerbates mastitis progression, with STH mammary tissue exhibiting adaptive responses through dynamic regulation of gene expression and metabolic reprogramming. This shift carries dual clinical implications: on one hand, it may represent an active protective measure by the host to prevent excessive inflammation and tissue damage; on the other hand, it clearly indicates the exhaustion of cellular repair mechanisms and the initiation of programmed cell death pathways. This directly explains why persistent infection leads to severe mammary tissue destruction and a significant, often irreversible, decline in lactation function. Notably, these transcriptional patterns show significant conservation across physiological states and species, aligning with transcriptomic profiles from ovine mammary tissue during lactation cycles [1] and mirroring observations in bovine models [36]. The temporal GO enrichment analysis reveals critical pathways underlying mastitis pathogenesis, including oxidative stress responses, metabolic shifts, and immune regulation. These results not only deepen our understanding of host–pathogen interactions but also identify promising molecular targets for developing novel diagnostic biomarkers, therapeutic interventions, and preventive strategies against mastitis.

KEGG pathway analysis revealed 22 conserved immune- and apoptosis-related signaling pathways that were consistently activated throughout *S. aureus* infection, with the MAPK signaling pathway emerging as the most significantly enriched (28 DEGs: *RASA1*, *CIB1*, *SHF*, *FLNA*, *ATF4*, *HSPB1*, *RASGRP2*, *TBC1D9B*, *COPG1*, *YLPM1*, *CHP1*, *RASAL3*, *MAP2K2*, *ARHGAP30*, *HSPA8*, *RPS6KA1*, *PAK2*, *NFATC3*, *ATF2*, *CACNA1H*, *SOS1*, *PROCA1*, *PPM1A*, *TGFBR*1, *ARHGAP21*, *CRK*, and *HSPA1B)*. Notably, RPS6KA1—a serine/threonine kinase and key *ERK1/2* substrate—exhibited sustained upregulation at 24 h, 48 h, and 72 h post-infection. As a critical MAPK effector, *RPS6KA1* regulates cellular proliferation, adhesion, and survival by phosphorylating downstream targets [37]. The MAPK pathway’s pivotal role in *S. aureus* inflammation is underscored by its dual capacity to (1) activate AP-1 transcription factors and (2) synergize with NF-κB/p65 signaling to amplify pro-inflammatory cytokine production [38]. This cascade underlies the classical clinical signs of mastitis, including localized redness, swelling, heat, and pain in the mammary gland. Mechanistically, *S. aureus* triggers TLR2/MyD88-dependent activation of both MAPK and NF-κB cascades, driving cytokine release and inflammatory tissue damage, as evidenced by elevated MAPK phosphorylation in infected uterine tissues [39,40]. These findings position MAPK signaling as a central coordinator of host–pathogen interactions, offering potential therapeutic targets for mitigating *S. aureus* mastitis [41].

Based on the cluster heatmap analysis (Figure 4), we observed that DEGs exhibited distinct temporal gradient characteristics: gene expression changes were relatively modest at 24 h post-infection, while significant differential expression emerged at 48 and 72 h. This time-dependent expression pattern provides crucial insights for elucidating the key regulatory networks during *S. aureus* infection. Protein–protein interaction (PPI) network analysis identified a core regulatory network of 120 interacting nodes, with PTK2B, UBC, STAT3, JAK1, and JAK3 emerging as central hub genes. Notably, PTK2B—a protein tyrosine kinase family member—showed significant downregulation, consistent with its established regulatory role in bovine mastitis pathogenesis [41]. Functional enrichment analysis demonstrated that these hub genes were predominantly involved in JAK-STAT and PI3K-Akt signaling pathways, aligning with previous findings in high-yield sheep where these pathways regulate milk synthesis [36]. These results collectively indicate that *S. aureus* infection disrupts mammary function through coordinated mechanisms including TLR2/MyD88-mediated innate immune activation, enhanced pro-inflammatory cytokine production (IL-6, TNF-α), and dysregulation of lactation-related pathways (JAK-STAT, PI3K-Akt). Furthermore, we observed differential expression of immune-related genes, with *CASP8* (apoptosis initiation) and *CCL19* (chemokine signaling) being upregulated, while *GCLC* (oxidative stress response) and *IL6R* (cytokine signaling) were downregulated, suggesting complex modulation of immune responses and cellular apoptosis during infection.

To further validate whether *S. aureus* infection activates the host innate immune response in STH mammary tissue, we performed RT-qPCR analysis of Toll-like receptor (TLR) expression. Our results revealed a significant parabolic increase in *TLR2*, *TLR4*, *TLR7*, and *TLR10* expression following infection, with *TLR2*—the primary receptor for *S. aureus* recognition—showing the most pronounced response. As the most well-characterized bacterial pattern recognition receptor in the TLR family, TLR2 mediates immune activation through regulation of key downstream effectors including MAPK, AP-1, and NF-κB signaling pathways [42]. In contrast to the upregulated *TLR2/4/7/10*, *TLR3* expression was significantly suppressed following *S. aureus* infection [43]. Although TLR3 is classically associated with antiviral defense and pro-inflammatory cytokine induction [44], emerging studies reveal broader functional roles, including recognition of endogenous nonviral ligands [45,46], initiation of anti-inflammatory signaling cascades, and fine-tuning of inflammatory responses [47,48,49]. However, the precise mechanisms by which TLR3 mediates immunoregulation during bacterial infections remain unclear and require further investigation, particularly in the context of mammary gland immunity.

Parallel analysis of key pro-inflammatory mediators (*IFN-α*, *IL-1β*, *IL-16*, *NF-κB*, and *TNF-α*) revealed a conserved temporal expression pattern characterized by peak levels at 36 hpi, followed by progressive decline. This temporal profile closely correlates with the clinical symptoms of mastitis: The upregulation of TLR2 and other receptors, along with the surge in pro-inflammatory mediators, directly triggers acute inflammatory responses in the mammary gland, manifesting as redness, swelling, heat, and pain. Conversely, suppression of TLR3 may impair the regulation of inflammation, exacerbating tissue damage. Although the decline in inflammatory mediators may alleviate acute symptoms, it is often accompanied by persistent impairment of mammary function.

## 5. Conclusions

Transcriptomic analysis of *S. aureus*-infected STH mammary tissue identified 1299 differentially expressed genes (DEGs) across three infection time points (24 h, 48 h, and 72 h), including 75 core DEGs persistently expressed throughout infection. Functional enrichment analysis revealed 22 significantly enriched signaling pathways associated with inflammation and apoptosis, while protein–protein interaction network analysis identified 120 interacting nodes with *PTK2B*, *UBC*, *STAT3*, *JAK1*, and *JAK3* as central hub genes. Furthermore, we observed substantial upregulation of key inflammatory mediators (*IFN-α*, *IL-1β*, *IL-16*, *NF-κB*, and *TNF-α*) and Toll-like receptors, demonstrating robust activation of innate immune responses during *S. aureus* infection.

As described above, upregulation of IL-1β, TNF-α, IL-16, and Toll-like receptor factors triggers the JAK-STAT signaling cascade. Concurrently, elevated expression of the apoptosis-related genes caspase-8 (cas8) and APOE activates apoptotic pathways, while the activation of metabolic pathways collectively drives the onset and progression of mastitis. These results collectively demonstrate that *S. aureus* infection induces mastitis through three interconnected mechanisms: (1) coordinated activation of innate and adaptive immune responses, (2) progressive disruption of mammary epithelial function, and (3) metabolic reprogramming of mammary tissue. Our study provides novel insights into the pathogenesis of mastitis while establishing a comprehensive conceptual framework for developing innovative therapeutic interventions and preventive strategies against ruminant mastitis.

## Figures and Tables

**Table 1:** The rating of the symptoms of STHs when challenged by *S. aureus*.

Symptom Rate Time	Activity	Feed and Water Intake	Fever	The Degree of Swelling	The Amount of Milk	The Color of Milk	The Consistency of Milk
24 h	+++	+++	++	+	+++	+	+
48 h	++	++	+++	++	++	++	++
72 h	++	++	+++	+++	+	++	++

Note: + represents weak, ++ represents moderate and +++ represents severe.

## Data Availability

The datasets presented in this study can be found in online repositories. The names of the repository/repositories and accession numbers can be found below: https://www.ncbi.nlm.nih.gov/bioproject/PRJNA778892/ (9 November 2021).

## Supplementary Materials

The following supporting information can be downloaded at https://www.mdpi.com/article/10.3390/pathogens14111133/s1.
