# Supplementary material for: Staphylococcus aureus Mastitis: A Time-Course Transcriptome of Immune Activation in Small-Tailed Han Sheep
Source: Pathogens. 2025 Nov 7;14(11):1133. doi: 10.3390/pathogens14111133 (PMC12655720; doi:10.3390/pathogens14111133)
Supplement: Supplementary file 1 [file pathogens-14-01133-s001.zip › pathogens-3880024-supplementary.pdf]

The response of small-tailed Han sheep to *Staphylococcus aureus* infection

Table S1. The summary of the RNA-Seq data.

Samples	Raw_reads	Clean_reads	Raw_Q20（%）	Clean_Q20（%）	Raw_Q30（%）	Clean_Q30（%）
Control	58650180	56696470	94.13%	94.61%	88.77%	89.28%
24h	54790268	52631676	95.47%	95.51%	90.56%	90.54%
48h	49668944	47984998	96.16%	96.16%	91.62%	91.55%
72h	46463356	45485796	93.48%	93.59%	87.59%	87.67%

Note: Differentially expressed genes (DEGs) at the peak of lactation and in non-lactating mammary gland parenchyma.

Table S2. The information of primers used in RT-PCR

引物名称<br>*Primer name/Gene*	引物序列（5'—3'）<br>*Primer Sequence*	产物大小/bp<br>*Produce size*	登录号<br>*Accession*
**TLR1 F:**	*CGTCTTTGCACCCAACAA*	*268*	*AY957612*
**TLR1 R:**	*ATTGCAGATGGACCTAGA*		
**TLR2 F:**	*ATGGGTGGTTCTTACCCG*	*173*	*NM001048231*
**TLR2 R:**	*AGTGCCTGCTGTCCCTGT*		
**TLR3 F:**	*GAGTTCGGAAGACGATGA*	*262*	*AY957614*
**TLR3 R:**	*GACAATAATGAAAGGGAC*		
**TLR4 F:**	*ACTGACGGGAAAGGGTATCC*	*209*	*AY958615*
**TLR4 R:**	*CAGGTTGGGAAGGTCAGTTT*		
**TLR5 F:**	*GTGGTAAACCTCGCCTAC*	*177*	*AY958616*
**TLR5 R:**	*ATCCCAATGTGATTCAAC*		
**TLR6 F:**	*ATTCAAATGCCCACCTTC*	*208*	*AY958617*
**TLR6 R:**	*TAGGACGTCACAAGATACTT*		
**TLR7 F:**	*GCTTTGGGAGTTGCGACAT*	*159*	*AY958618*
**TLR7 R:**	*ACCCACCAGACTTTCCACA*		
**TLR10 F:**	*TCCCATACATCAGTAGAGT*	*242*	*AY958621*
**TLR10 R:**	*GATACATACCCAACAGCA*		
**β-actin F:**	*TCTTCCAGCCTTCCTTCCTG*	*108*	*U3935*
**β-actin R:**	*GAAGGCGTAGAGGTCTTTGC*		
**TNF-α F**	*TCTCAAGCCTCAAATAACAA*	*172*	*EF446377.1*
**TNF-α R**	*TCTAAGTATCCTCCACCCTC*		
**IL-16 F**	*AGTGAAGACCCCGCAAACA*	*116*	*XM-004017797.3*
**IL-16 R**	*GCAAGGAGATAACCGACTGACC*		
**IL-1β F**	*TCTCCCTAAAGAAGCCATAC*	*153*	*NM001009465*
**IL-1β R**	*AGAGCGTCTCAGCACGAATA*		
**IFN-α F**	*AACCTCCCAGGCACA*	*164*	*HQ585519*
**IFN-α R**	*TGGATCAGCAGCTCACT*		
**NF-κB F**	*GCATTCTTTGTGCCTATC*	*169*	MT799836.1
**NF-κB R**	*AAGGAGGAACAGAGCAGT*		
**Casp8 F**	*GCCTGATTGCTACTGATG*	*114*	*XM_042244960.2*
**Casp8 R**	*AAGTCTAAAGAGTGGTGGT*		
**CCL19 F**	CCCTGTATTCCTGGTGCG	195	XM_004004120.5
**CCL19 R**	GGATGTCATTGGGTAACTGC		
**GCLC F**	CCCCGTCTTGAACTCCTA	*146*	*XM_015102644.4*
**GCLC R**	*GCTTTGCGATAAACTCCC*		
**IL-6R F**	*TGGGTAAAGAACGCAAAG*	*138*	*NM_173923.2*
**IL-6R R**	*CTGACCAGAGGAGGGAAT*		

Table S3. Common DEGs at 24h, 48h, and 72h

GeneID	GeneName	Up/down	Description
ENSOARG00000001267	-	down	Peptidyl-prolyl cis-trans isomerase
ENSOARG00000001198	-	up	
ENSOARG00000002384	-	up	Uncharacterized protein
ENSOARG00000002892	-	up	Eukaryotic translation initiation factor 3 subunit C
ENSOARG00000003068	-	up	Caspase
ENSOARG00000003957	-	up	60S ribosomal protein L13
ENSOARG00000007439	-	up	
ENSOARG00000011154	-	up	Uncharacterized protein
ENSOARG00000012450	-	up	Ferritin
ENSOARG00000014790	-	up	Clusterin
ENSOARG00000016241	-	up	Uncharacterized protein
ENSOARG00000016908	-	up	Uncharacterized protein
ENSOARG00000017320	-	up	Uncharacterized protein
ENSOARG00000017679	-	up	Ferritin
ENSOARG00000017744	-	up	Uncharacterized protein
ENSOARG00000018207	-	up	Uncharacterized protein
ENSOARG00000025846	-	up	-
ENSOARG00000017954	ACSL4	up	acyl-CoA synthetase long-chain family member 4
ENSOARG00000005791	ACTN4	up	actinin, alpha 4
ENSOARG00000004143	ALDOA	up	aldolase, fructose-bisphosphate A
ENSOARG00000009438	APOE	up	apolipoprotein E
ENSOARG00000015056	APP	up	amyloid beta (A4) precursor protein
ENSOARG00000018617	ATRX	up	alpha thalassemia/mental retardation syndrome X-linked
ENSOARG00000007183	BAG6	up	BCL2 associated athanogene 6
ENSOARG00000007922	BCL7C	up	B-cell CLL/lymphoma 7C
ENSOARG00000017454	SMARCA4	up	SWI/SNF related, matrix associated, actin dependent regulator of chromatin, subfamily a, member 4
ENSOARG00000016922	CASP8	up	caspase 8
ENSOARG00000015275	CCDC66	up	coiled-coil domain containing 66
ENSOARG00000016476	COL3A1	up	collagen, type III, alpha 1
ENSOARG00000011109	EEF2	up	eukaryotic translation elongation factor 2
ENSOARG00000020596	EIF4G1	up	eukaryotic translation initiation factor 4 gamma, 1
ENSOARG00000013400	EIF5B	up	eukaryotic translation initiation factor 5B
ENSOARG00000011387	ENG	up	endoglin
ENSOARG00000009659	EPHX2	up	epoxide hydrolase 2, cytoplasmic
ENSOARG00000014970	FAM20A	up	family with sequence similarity 20 member A
ENSOARG00000020518	GGCX	up	gamma-glutamyl carboxylase
ENSOARG00000007288	GNB2L1	up	guanine nucleotide binding protein (G protein), beta polypeptide 2-like 1
ENSOARG00000002820	HCK	up	HCK proto-oncogene, Src family tyrosine kinase
ENSOARG00000017375	HIPK3	up	homeodomain interacting protein kinase 3
ENSOARG00000004985	HNRNPU	up	heterogeneous nuclear ribonucleoprotein U (scaffold attachment factor A)

ENSOARG00000003589	HOOK3	up	hook microtubule-tethering protein 3
ENSOARG00000009343	HSP90AB1	up	heat shock protein 90kDa alpha family class B member 1
ENSOARG00000007124	IKBKAP	up	inhibitor of kappa light polypeptide gene enhancer in B-cells, kinase complex-associated protein
ENSOARG00000017187	KRT7	up	keratin 7, type II
ENSOARG00000002094	LAPTM5	up	lysosomal protein transmembrane 5
ENSOARG00000020958	LEO1	up	LEO1 homolog, Paf1/RNA polymerase II complex component
ENSOARG00000007337	MTCH2	up	mitochondrial carrier 2
ENSOARG00000005012	NBR1	up	neighbor of BRCA1 gene 1
ENSOARG00000009782	NDUFS2	up	NADH:ubiquinone oxidoreductase core subunit S2
ENSOARG00000011923	NECAP2	up	NECAP endocytosis associated 2
ENSOARG00000020442	NOTCH2	up	notch 2
ENSOARG00000010167	NR2F2	up	nuclear receptor subfamily 2 group F member 2
ENSOARG00000004262	NUCKS1	up	nuclear casein kinase and cyclin-dependent kinase substrate 1
ENSOARG00000004501	PCSK7	up	proprotein convertase subtilisin/kexin type 7
ENSOARG00000011864	PFKL	up	phosphofructokinase, liver
ENSOARG00000006032	PHB	up	prohibitin
ENSOARG00000005558	PKN1	up	protein kinase N1
ENSOARG00000000605	PLXNB1	up	plexin B1
ENSOARG00000017998	PPP1R7	up	protein phosphatase 1 regulatory subunit 7
ENSOARG00000007622	PSMB8	up	proteasome subunit beta 8
ENSOARG00000019212	PSME2	up	proteasome activator subunit 2
ENSOARG00000000683	PTP4A2	up	protein tyrosine phosphatase type IVA, member 2
ENSOARG00000011244	RDH5	up	retinol dehydrogenase 5 (11-cis/9-cis)
ENSOARG00000002624	RPS27A	up	ribosomal protein S27a
ENSOARG00000004384	RPS6KA1	up	ribosomal protein S6 kinase, 90kDa, polypeptide 1
ENSOARG00000011653	RXFP2	up	relaxin/insulin-like family peptide receptor 2
ENSOARG00000006080	SDF4	up	stromal cell derived factor 4
ENSOARG00000011282	SERBP1	up	SERPINE1 mRNA binding protein 1
ENSOARG00000016653	SFXN1	up	sideroflexin 1
ENSOARG00000014810	SMARCE1	up	SWI/SNF related, matrix associated, actin dependent regulator of chromatin, subfamily e,
ENSOARG00000009032	SPARC	up	secreted protein, acidic, cysteine-rich (osteonectin)
ENSOARG00000000491	SRRM2	up	serine/arginine repetitive matrix 2
ENSOARG00000011887	SRSF11	up	serine/arginine-rich splicing factor 11
ENSOARG00000019668	THRAP3	up	thyroid hormone receptor associated protein 3
ENSOARG00000002015	XAB2	up	XPA binding protein 2
